# Formylation of Amines

**DOI:** 10.3390/molecules19067689

**Published:** 2014-06-10

**Authors:** Ciera J. Gerack, Lisa McElwee-White

**Affiliations:** Department of Chemistry, University of Florida, Gainesville, FL 32611-7200, USA; E-Mail: cjgerack@gmail.com

**Keywords:** carbonylation, formylation, amines

## Abstract

Methods to convert amines to formamides are of interest due to the many uses of formamides as synthetic intermediates. These methods include stoichiometric reactions of formylating reagents and catalytic reactions with CO as the carbonyl source. This review discusses the reported stoichiometric and catalytic approaches for preparation of formamides.

## 1. Introduction

Formamides are an important class of compounds that appear as intermediates in fungicide [1,2] and pharmaceutical syntheses [1,3,4,5], and isocyanate [6], formamidine [7], and nitrile formation [8]. Formamides also serve as reagents in functional group conversion [9], the Vilsmeier formylation reaction [10], and the allylation [11] and hydrosilation [12] of carbonyl compounds. Due to their wide range of applications, many approaches have been developed to synthesize formamides. The methods discussed in this review include the use of stoichiometric formylating agents, acid catalysts, organic catalysts, transition metal catalysts, and catalytic carbonylation.

## 2. Stoichiometric Formylating Agents

**Scheme 1:**
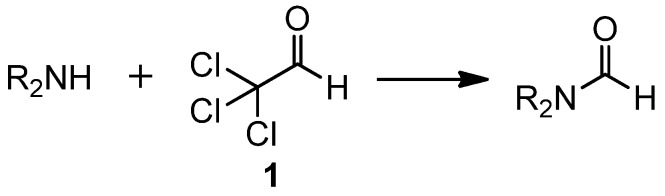
Formylation using **1**.

The earliest methods for formylation of amines involved formylating agents such as chloral, formic acid, formaldehyde, and formates. In 1952, Blicke reported the formylation of amines with chloral (**1**) (Scheme 1) [13]. This method produced excellent yields at low temperature, producing only chloroform as a byproduct. Successful substrates for this method include strongly basic primary amines, diamines, cyclic secondary amines, and sterically hindered secondary amines.

**Scheme 2:**
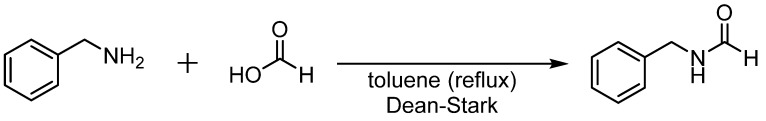
Formylation of amines using formic acid.

Formic acid itself can be used to achieve formylation by dehydration (Scheme 2) [14]. The amine and formic acid were dissolved in toluene and the solution was refluxed in the presence of a Dean-Stark trap, which collected the water produced by the condensation reaction. 

An example of *N*-formylation by formic acid under solvent-free conditions was reported by Hajra [15]. The amine and formic acid were heated to 80 °C until the reaction reached completion. Formamide products were obtained in good to excellent yields from substituted aromatic amines as well as primary and secondary alkyl amines. The yields produced by aliphatic amines yields were lower than those from aromatic amines. When a mixture of primary and secondary amines was exposed to the reaction conditions, the primary amines were formylated selectively. Hydroxyl substituents remained intact after formylation of the amine and no isolable side products were observed.

**Scheme 3:**
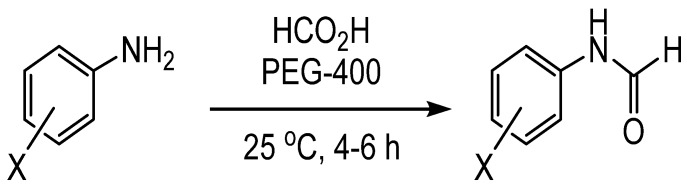
Formylation of aromatic amines using formic acid and polyethylene glycol.

Formic acid in polyethylene glycol has been shown to formylate anilines (Scheme 3) [16]. This reaction can be carried out at room temperature under an inert atmosphere in relatively short reaction times of 4–6 h. The conditions are tolerant of functional groups such as nitro, halogen, ester, ketone, and alkyl groups. Attempts to formylate the oxygen of phenols with these conditions were unsuccessful, presumably due to the lower nucleophilicity of the phenol.

**Scheme 4:**
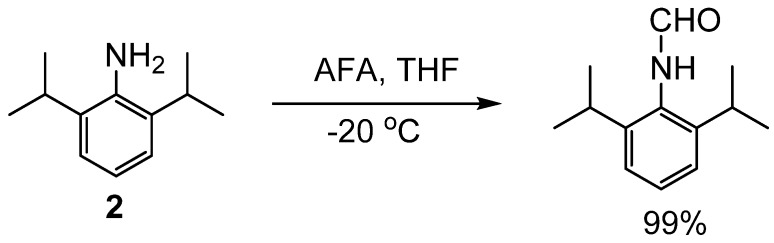
Formylation of a sterically hindered amine with AFA.

There are many examples of *N*-formylation by acetic formic anhydride (AFA) [17,18,19,20,21,22,23]. One such example is a one-pot procedure for *N*-monomethylation of primary amines that proceeds through *N*-formylation followed by reduction [24]. Amines were allowed to react with AFA, which was generated *in situ* from excess formic acid and acetic anhydride at −20 °C (Scheme 4). The reaction reached completion for most amines in less than 15 min and the resulting formamides were isolated in yields of 97%–100%. High yields were achieved for formylation of simple alkyl, aromatic, multifunctional, and sterically hindered amines, such as **2**.

Formylation is often used as a means of protecting amino groups in peptide synthesis. As early as 1932, du Vigneaud had reported a procedure for *N*-formylating amino acids with formic acid and acetic anhydride [25]. This method was used as a way to protect the amines during resolution of d,l-cystine by selective crystallization of the strychnine salt. This method of formylating amino acids was applied by Yang as a means of protecting the amino group of many additional amino acids [26]. When the amino acids were exposed to the reaction conditions, the procedure yielded the *N*-formyl aminoacids in yields of 78%–90%.

Problems with racemization of *tert*-butyl esters of amino acids during attempted formylation with by AFA-were addressed using a modification that allows the formylation of *tert*-butyl amino acid esters with minimal or no racemization [27]. This method combined formic acid with dicyclohexyl-carbodiimide (DCC) to form the active formylating reagent, which was added to solutions of *tert*-butyl amino acid esters. The protected amino acid esters were produced in high yields. A related method of formylating amino acid esters was reported by Benoiton [28]. In this method *N*-ethyl-*N*′-(3-dimethylaminopropyl)-carbodiimide is used to prepare the formic anhydride which is then allowed to react with the ester salt in the presence of *N*-methylmorpholine. This method does not require any specialized purification and produced high yields from methyl, benzyl and *tert*-butyl esters. Formylation of amino acid esters can also be carried out employing cyanomethylformate as a formylating agent [29]. This method was successfully applied to methyl, ethyl, benzyl, and *tert*-butyl amino acid esters and afforded good to excellent yields without loss of optical purity.

**Scheme 5:**
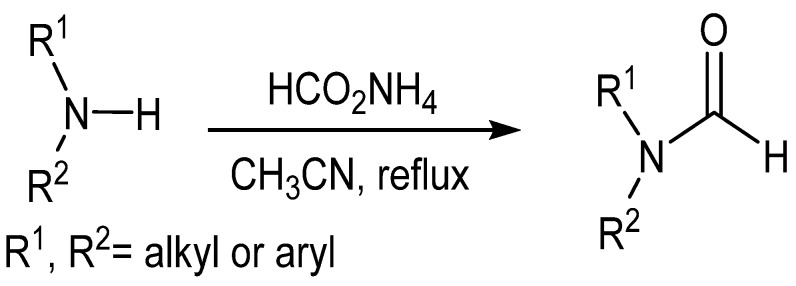
General formylation by formic acid in ammonium formate.

Ammonium formate has been shown to formylate both anilines and secondary amines in good to excellent yields upon reflux in acetonitrile (Scheme 5) [30]. With the exception of benzylamine, primary amines produced alkyl formate salts instead of the expected formamide products.

**Scheme 6:**
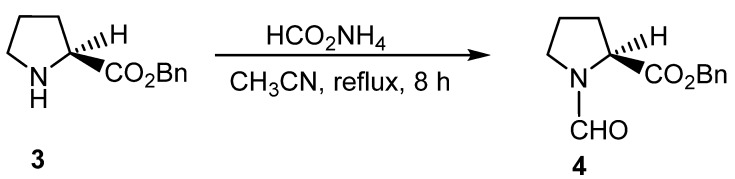
Formylation of **3** without racemization of the chiral center.

These conditions are also applicable as protecting groups for chiral molecules. The benzyl ester of l-proline (**3)** was successfully formylated to **4** in 75% yield without any observed racemization (Scheme 6). When hydroxyl groups were present, ammonium formate selectively formylated the nitrogen, leaving the hydroxyl group intact.

**Scheme 7:**
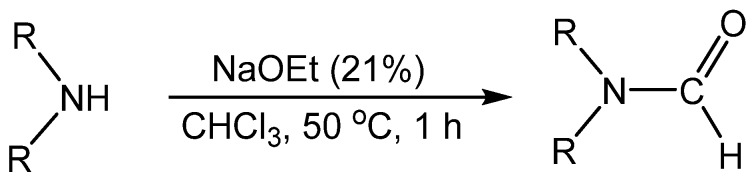
*N*-formylation of secondary amines.

**Scheme 8:**
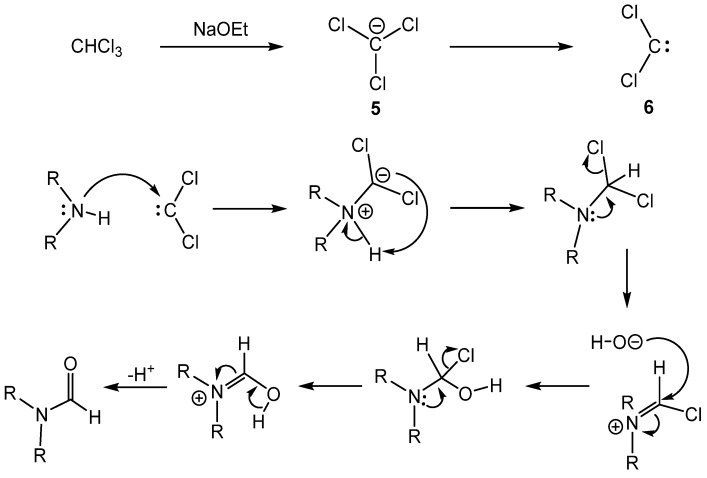
General mechanism of the formylation via the Reimer-Tiemann reaction. Scheme adapted from reference [31].

A recent method used the Reimer-Tiemann (R-T) reaction to produce formamides from secondary amines (Scheme 7) [31]. Alkyl, cyclic, and *N*-methylaniline derivatives all produced formamides in good to excellent yields, but the best yields were obtained with cyclic amines. A mechanistic pathway consistent with the R-T reaction was proposed (Scheme 8). First, chloroform reacted with sodium ethoxide to form the trichloromethyl carbanion **5**, which readily loses chloride to generate dichlorocarbene **6**. Then carbene **6** reacted with the amine and produced the formamide product through the R-T reaction.

## 3. Catalytic Formylation with Acid Catalysts

**Scheme 9:**
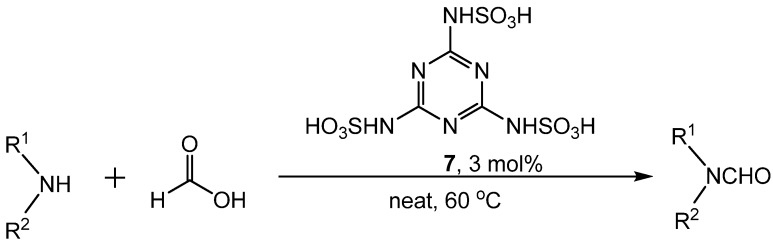
General reaction of MTSA catalyzed formylation.

**Scheme 10:**
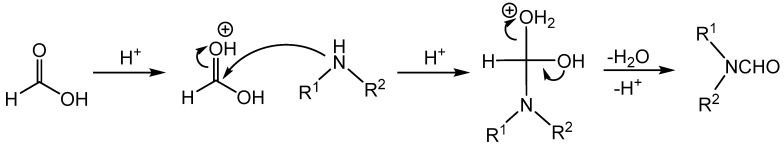
Proposed reaction mechanism for MTSA catalyzed formylation.

In a procedure reported by Yang, melaminetrisulfonic acid (MTSA, **7**) catalyzed formylation with formic acid in solvent-free conditions (Scheme 9) [32]. The amine, two equivalents of formic acid, and 3 mol% **7** were stirred at 60 °C until completion of the reaction (40–90 min). Substituted aniline derivatives were examined and all produced excellent yields of formamides, regardless of the presence of electron donating or electron withdrawing substituents. Primary and secondary amines also produced formamides in high yields. A proposed mechanism suggests that formic acid is protonated by **7**, followed by nucleophilic attack of the amine. Subsequent elimination of water produces the formamide (Scheme 10).

**Scheme 11:**
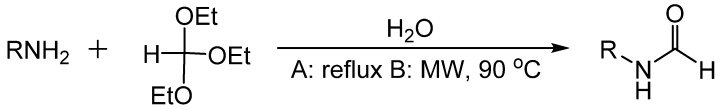
Reaction of primary amines with triethyl orthoformate in water.

Roberts initially reported that amines and triethyl orthoformate produced the corresponding *N*-ethyl formamides in the presence of sulfuric acid at high temperature [19]. Swaringen performed a similar transformation using *p*-toluenesulfonic acid and triethyl orthoformate [33]. Using a similar method, Kaboudin reported simple *N*-formylation without an alkyl shift onto the nitrogen (Scheme 11) [34]. In this method, formylation of primary amines took place in water with triethyl orthoformate in the absence of base, acid, or catalyst in moderate to good yields. No results from secondary amines were reported. Water proved to be the optimal solvent after experiments with ethanol, ethyl acetate, dichloromethane (DCM), chloroform, dimethylformamide (DMF), and dimethylsulfoxide (DMSO). Product could obtained either by reflux in water for 24–48 h (method A) or by microwave irradiation at 90 °C for 2–3 h (method B). 

Formic acid and a catalytic amount of sodium formate have been reported to produce formamides from amines at room temperature under solvent-free conditions [35]. Functionalized anilines, primary amines, cyclic secondary amines, and sterically hindered secondary amines all produced good to excellent yields of formamides in less than 8 h. The reaction was selective for *N*-formylation over *O*-formylation in the presence of unprotected hydroxyl groups on the amine substrates. The sodium formate used in the reaction could be isolated from the reaction mixtures and reused up to four times without a loss of activity.

**Scheme 12:**
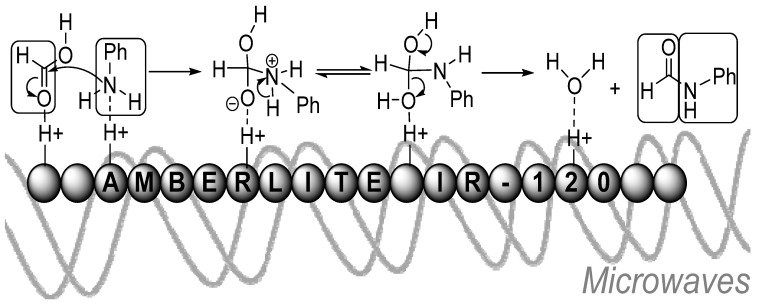
Amberlite IR-120 catalyzed formylation. Scheme adapted from reference [36].

*N*-formylation of anilines and simple primary amines by formic acid can also be carried out in solvent-free conditions with the reusable ion exchange resin Amberlite IR-120[H^+^] as an acid catalyst [36]. Mixtures of Amberlite IR-120, the amine and formic acid were exposed to microwave irradiation for 20 s intervals until all starting material was consumed. The reactions were complete between 60–120 s regardless of amine or substituents and the formylated amines were obtained in excellent yields. At the end of each reaction, the resin was easily isolated and was reusable up to five times without a loss in activity. The proposed mechanistic pathway involved coordination to the resin through hydrogen bonds (Scheme 12), followed by attack at the carbonyl by the amine. Subsequent rearrangement produced the formamide, leaving water coordinated to the resin.

**Scheme 13:**
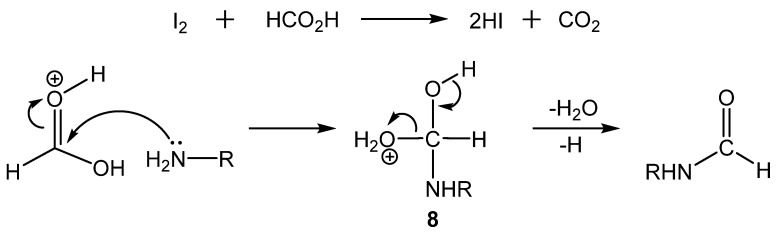
Mechanism of I_2_ catalyzed formylation.

Jang reported *N*-formylation in solvent-free conditions with molecular iodine (I_2_) as a catalyst [37]. During optimization studies, it was determined that aniline, 5 mol% I_2_, and two equivalents of formic acid produced formanilide in excellent yield after 2 h at 70 °C. After optimization was completed, several aniline derivatives as well as primary and secondary amines were subjected to the reaction conditions and produced formamides in good to excellent yields. As it is known that I_2_ reacts with formic acid to produce HI [38], it was assumed that HI was the active catalytic species, generated *in situ*. In the proposed mechanism, protonated formic acid is attacked by the amine, followed by proton transfer to provide **8**, and finally through elimination of water and a proton, the formamide is formed (Scheme 13).

**Scheme 14:**
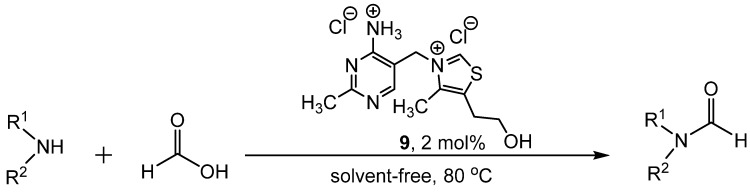
Solvent-free formylation catalyzed by **9**.

**Scheme 15:**
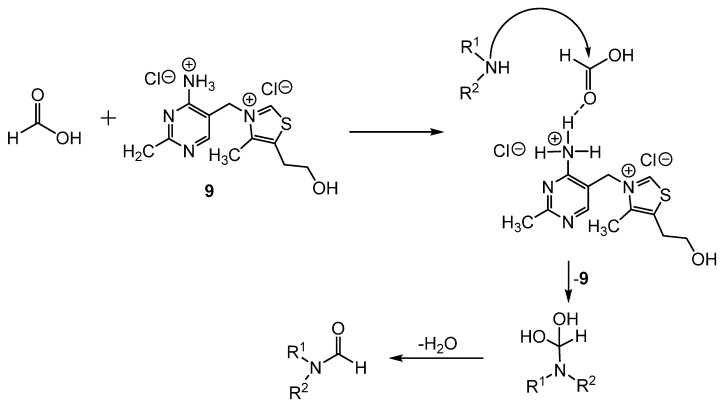
Proposed mechanistic pathway for formamide synthesis catalyzed by **9**.

Hu reported the use of thiamine hydrochloride **9** as a catalyst to produce formamides from the amine and formic acid in solvent-free conditions (Scheme 14) [39]. This method was successfully applied to aromatic and aliphatic amines, with yields ranging from 88%–96%. When other carboxylic acids were used in place of formic acid, the corresponding amides were produced. While the mechanistic pathway is not known, it was suggested that the catalyst activates formic acid through hydrogen bonding. After nucleophilic attack of the amine at the carbonyl of formic acid, the formamides were produced through the elimination of water (Scheme 15).

**Scheme 16:**
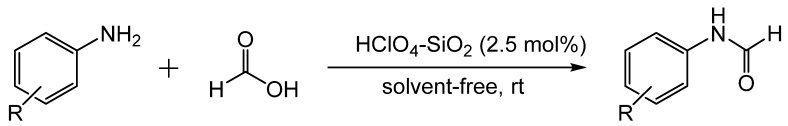
Silica supported acid catalyzed *N*-formylation.

Other solvent-free, acid catalyzed methods have been reported. Hajela reported silica supported perchloric acid (HClO_4_-SiO_2_) catalyzed *N*-formylation of aromatic and cyclic secondary amines (Scheme 16) [40]. When substrates with hydroxyl groups were exposed to this reaction, formylation occurred selectively at the amino position. Other silica supported acids including sulfuric (H_2_SO_4_), fluoroboric (HBF_4_), and trifluoroacetic (TFA) acids were examined but all produced lower yields of products. The catalyst was easily removed at the completion of the reaction and after washing and drying, could be used up to three times without a loss in activity.

**Scheme 17:**
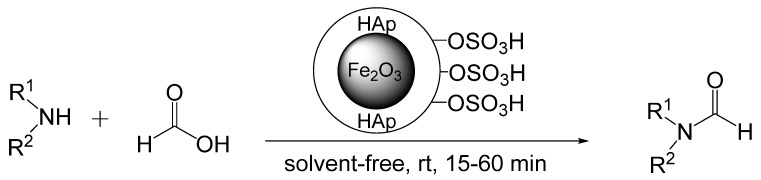
Formylation of amines by formic acid with a supported acid catalyst.

Sulfonic acid supported on hydroxyapatite (HAp)-encapsulated-γ-Fe_2_O_3_ nanocrystallites acts as a Br**ø**nsted acid and catalyzes formylation of aromatic, primary, and secondary amines with formic acid (Scheme 17) [41]. No *O*-formylation occurred on amines containing hydroxyl groups. Optimum conditions are amine, 1.2 equivalents of formic acid and 0.9 mol% SO_3_H (γ-Fe_2_O_3_@ HAp-SO_3_H) at room temperature. The reaction was monitored by TLC and required 15–60 min to reach completion. The magnetic solid-state catalyst was easily removed from the reaction mixtures by attaching an external magnet to the vessel and decanting the reaction solutions. After washing and drying the catalyst could be reused for four consecutive trials without a loss in activity. In order to ascertain whether protons lost from the particles were catalyzing the reaction homogenously, the reaction was performed for 10 min, the catalyst was removed by an external magnet, and the reaction was allowed to continue for 3 h. After removal of the catalyst, no additional product had formed.

**Scheme 18:**
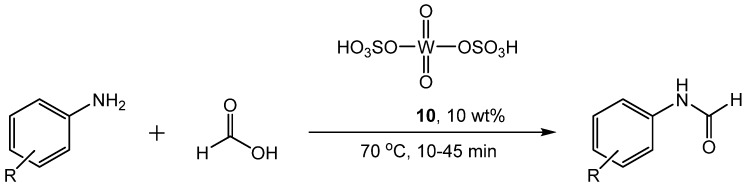
*N*-formylation of amines with formic acid and sulfated tungstate catalyst.

Another transition metal catalyst that acts as an acid was reported by Akamanchi [42]. Sulfated tungstate **10** catalyzed the reaction of amines with formic acid to produce formamides in solvent-free conditions (Scheme 18).

The optimized conditions are 10 mol% **10**, 70 °C and 1.2 equivalents of formic acid for 10–45 min. The catalyst was easily isolated after the reaction and could be reused up to four times without experiencing any loss of activity. Yields ranged from 85%–99% for formylation of primary, secondary, aromatic, heteroaromatic, and alkyl amines as well as α-amino acids. Studies of the interaction between the catalyst and the reagents indicated that formic acid was adsorbed onto the catalyst, but amine was not adsorbed. This suggests a mechanism in which the catalyst activates the formic acid followed by nucleophilic attack, similar to other acid catalyzed formylations.

## 4. Catalytic Formylation with Organic Catalysts

**Scheme 19:**
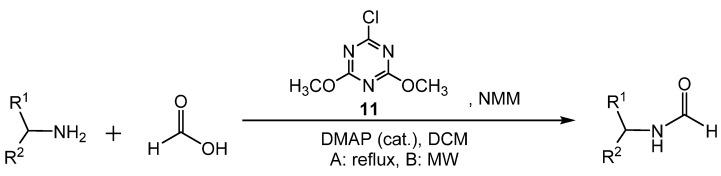
One step preparation of formamides by method A at reflux or method B via microwave irradiation.

**Scheme 20:**
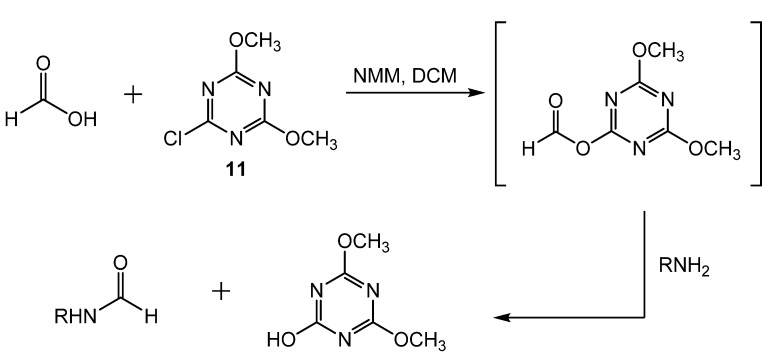
Pathway of formamide formation from formic acid and **11**.

Formylation with formic acid and 2-chloro-4,6-dimethoxy[1.3.5]triazine (CDMT, **11**) was reported by Giacomelli [43]. Amines and amino acid esters were formylated in nearly quantitative yields either in DCM at reflux (method A) or under microwave irradiation (method B) (Scheme 19) to yield the formamide products in a one pot process. In method A, dry formic acid and the amine were treated with **11** and 4-(dimethylamino)pyridine (DMAP) as a catalyst. *N*-methylmorpholine (NMM) and DCM were added, and the solution was refluxed. This reaction required 5–20 h to reach completion. However, with the use of microwave irradiation in method B, the reaction produced the formamides after only 3–6 min. When chiral amino acid esters were used, optical purity was maintained but steric hindrance in the amine resulted in slightly lowered yields. The proposed mechanistic pathway involved the formation of a formate ester intermediate composed of formic acid and **11**, which then is attacked by the amine to form the formamide (Scheme 20).

**Scheme 21:**
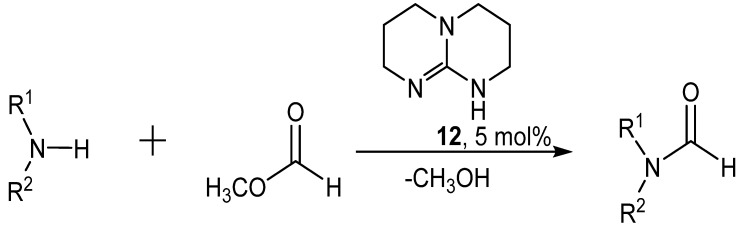
Formylation of amines by methyl formate and catalytic base.

Deutsch reported formylation of amines by methyl formate and catalytic base (Scheme 21) [44]. Amidine and guanidine catalysts were examined in the formylation of morpholine and *tert*-butylamine at room temperature. The best catalyst for this reaction was 1,5,7-triazabicyclo[4.4.0]dec-5-ene (TBD, **12**).

**Figure 1:**
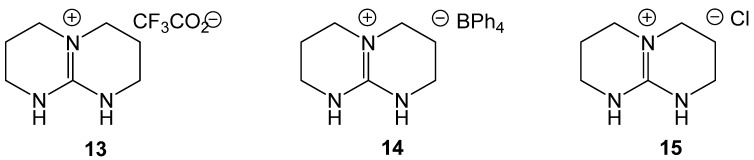
TBD based ILs examined for catalytic activity towards *N*-formylation.

**Scheme 22:**
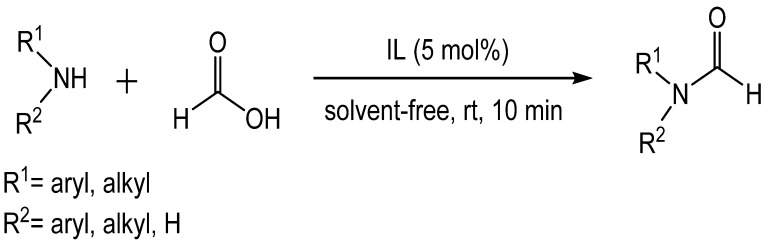
Optimized conditions for IL catalyzed formylation.

**Scheme 23:**
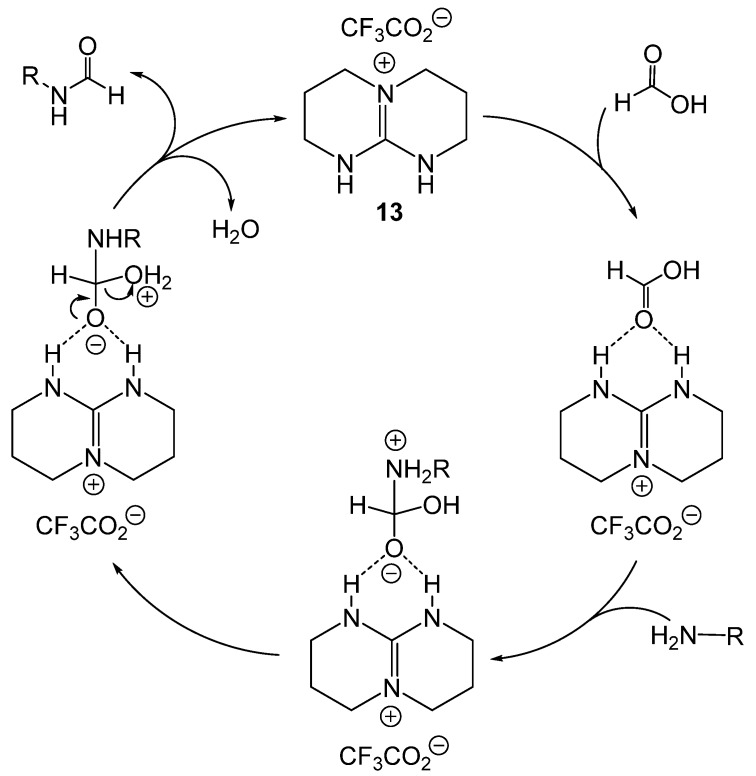
Proposed mechanism for IL-catalyzed formamide production.

Ionic liquids (IL) have recently been reported to catalyze formylation. ILs are attractive because of their stability, ease of removal, and easy synthesis. Baghbanian reported that amine, formic acid, and TBD based ionic liquids produced formamides from aromatic, alkyl, and heteroaromatic amines as well as amino alcohols in good to excellent yields (Figure 1) [45]. Three related ionic liquids were examined (**13**, **14**, and **15**) with **12** being preferred (Scheme 22). Addition of solvent to the reaction mixtures resulted in decreased yields. The IL was easily separated from the product and reused up to six times without a loss in activity. A proposed mechanism indicated that the reaction was mediated through hydrogen bonding with the catalyst (Scheme 23).

## 5. Catalytic Formylation with Metal Catalysts

**Scheme 24:**
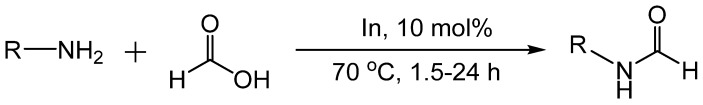
Indium catalyzed formylation of amines.

Metals have also been used as catalysts for formylation of amines using stoichiometric amounts of formic acid or another formylating agent. Jang reported solvent-free conditions in which amine, formic acid, and 10 mol% indium at 70 °C produced formamide in moderate to excellent yields (Scheme 24) [46]. Without indium, the yields were considerably lower. Aniline derivatives, primary amines, secondary amines, and amino alcohols were all successfully formylated under these conditions, with the reaction time varying between 1–24 h depending on the electronics and sterics of the amine. Amino groups of methyl and benzyl α–amino acid esters could be protected in good yields under these conditions with no racemization.

**Scheme 25:**
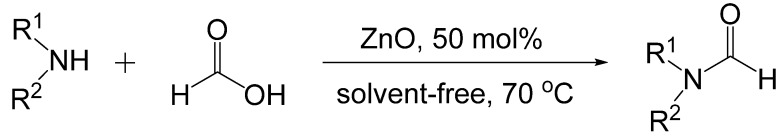
ZnO catalyzed formylation of amines with formic acid.

A variety of transition metal Lewis acids have also been used in *N*-formylation of amines. ZnO was reported by Hosseini-Sarvari as a Lewis acid catalyst for the solvent-free formylation of aromatic, primary and secondary amines with formic acid in good to excellent yield [47]. Optimal conditions for this reaction are 3 equivalents of formic acid, 50 mol% catalyst and 70 °C for 10–720 min (Scheme 25). The reaction could be scaled up from 1 mmol amine to 100 mmol amine without any loss of yield. The ZnO catalyst was filtered out of the reaction mixture at the completion of the reaction and washed with DCM, after which it could be successfully recycled up to three times. Longer reaction times were necessary for aromatic amines containing electron withdrawing groups as well as for secondary amines. When a mixture of primary and secondary amines was subjected to the reaction conditions, primary amines were preferentially formylated. Amines containing hydroxyl groups were selectively formylated at the amino group and competitive *O*-formylation was not observed.

**Scheme 26:**
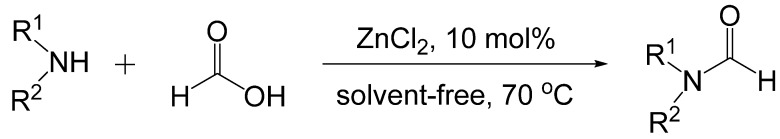
Solvent-free formylation of amines using ZnCl_2_ catalyst.

Similar Lewis acid (LA)-catalyzed, solvent-free conditions for formylation were reported by Rao [48]. A Lewis acid catalyst and formic acid were used to produce high yields of the desired formamide products (Scheme 26). Although Lewis acids such as FeCl_3_, AlCl_3_, and NiCl_2_ could be used, the inexpensive, environmentally friendly catalyst ZnCl_2_ produced the best results. The optimum conditions are 10 mol% catalyst, 3 equivalents of formic acid and 70 °C for 10–900 min. The reaction required longer reaction times for electron poor aromatic amines and secondary amines but tolerated a variety of functional groups such as nitro, halogen, ester, ketone, and alkyl. The proposed reaction mechanism is similar to other acid catalyzed reactions of formic acid and amines.

**Scheme 27:**
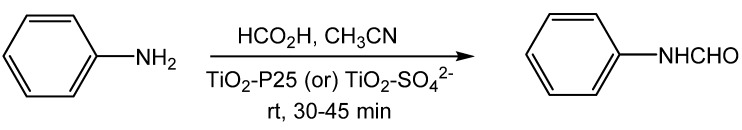
Amine formylation from formic acid catalyzed by TiO_2_-P25 or TiO_2_-SO_4_^2−^.

**Scheme 28:**
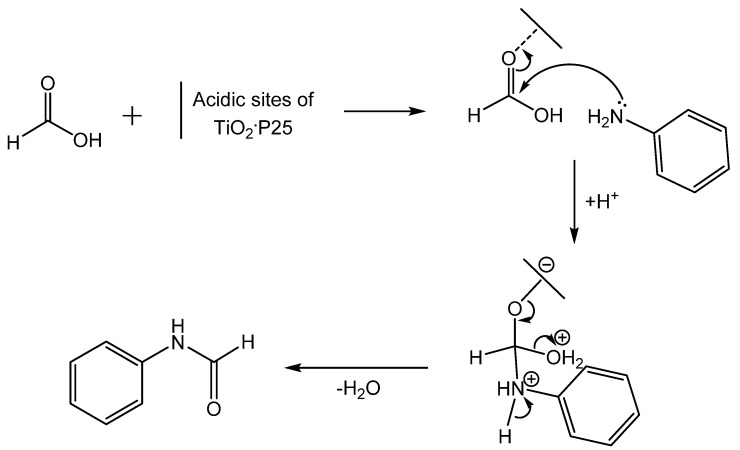
Mechanism of TiO_2_-P25 catalyzed formylation.

Formylation of amines with formic acid and the nanoparticle photocatalyst TiO_2_-P25 or sulfated titania was reported by Swaminathan as an extension of research on semiconductor photocatalysts [49]. In this work, either TiO_2_-P25 or TiO_2_-SO_4_^2−^ catalyzed formylation of amines with formic acid in short reaction times at room temperature (Scheme 27). This method was applied to substituted aromatic amines as well as primary and secondary aliphatic amines. In all cases studied, TiO_2_-SO_4_^2−^ produced better yields, ranging from moderate to excellent. In recyclability tests, TiO_2_-SO_4_^2−^ could be reused up to five times without a loss in activity, while TiO_2_-P25 suffered a 50% drop in activity during the second trial. A proposed mechanism invokes acid catalyzed formylation (Scheme 28). Lewis acidic sites on the catalyst coordinate formic acid and facilitate nucleophilic attack of the amine on the carbonyl. The formamide product is then produced by loss of water.

**Scheme 29:**
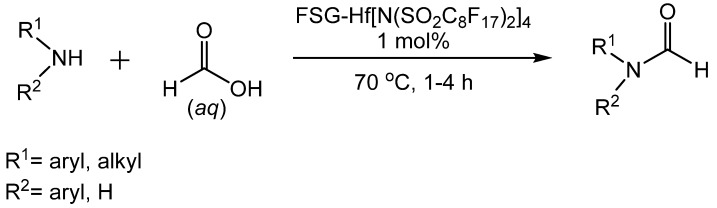
*N*-formylation catalyzed by FSG-Hf[N(SO_2_C_8_F_17_)_2_]_4_.

**Scheme 30:**
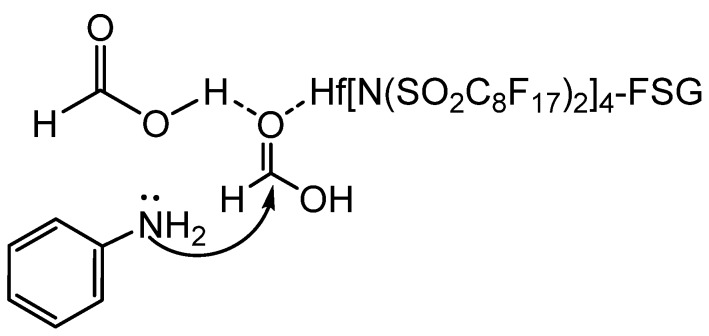
Electronic assistance for nucleophilic attack of amine on formic acid.

More recently, Hong reported that the fluorous silica gel-supported hafnium (IV)bis(perfluorooctanesulfonyl)imide complex (FSG-Hf[N(SO_2_C_8_F_17_)_2_]_4_)-catalyzed formylation of amines in aqueous formic acid (Scheme 29) [50]. Optimum conditions were 1 mol% catalyst, 70 °C, and 3 equivalents of formic acid. The catalyst could be reused for up to three cycles without loss of activity. Aromatic amines produced the desired formamides in high yields regardless of substituent. However, when electron withdrawing groups were present, longer reaction times were necessary. Aliphatic *N*-butylamine and secondary diphenylamine produced good yields. It was proposed that the high loading of formic acid on the catalyst resulted in hydrogen bonding that rendered the carbonyl more electrophilic and facilitated attack by the amine (Scheme 30).

**Scheme 31:**
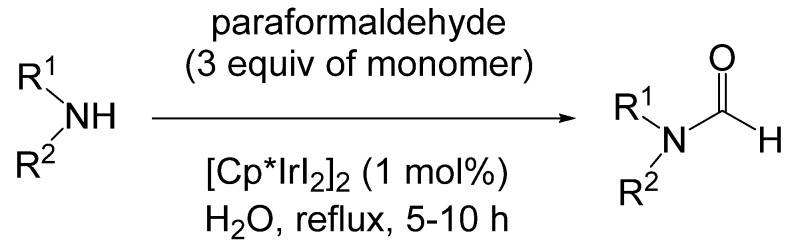
Iridium catalyzed formylation of amines with paraformaldehyde.

Williams reported *N*-formylation of amines with paraformaldehyde in the presence of an iridium catalyst [51]. Optimal conditions for this reaction were amine, paraformaldehyde (3 equivalents of the monomer) and 1 mol% [Cp*IrI_2_]_2_ as catalyst in refluxing water for 5–10 h (Scheme 31). Yields of the formamides were high for primary amines and moderate to excellent for secondary amines. When an enantiomerically pure amine was reacted, the formamide product retained most but not all of its enantiomeric purity. Primary anilines did not afford product under these conditions. The acyclic secondary aniline examined produced the formamide in only 46% yield, but indoline, a cyclic secondary aniline, produced the formamide in 91% yield.

**Scheme 32:**
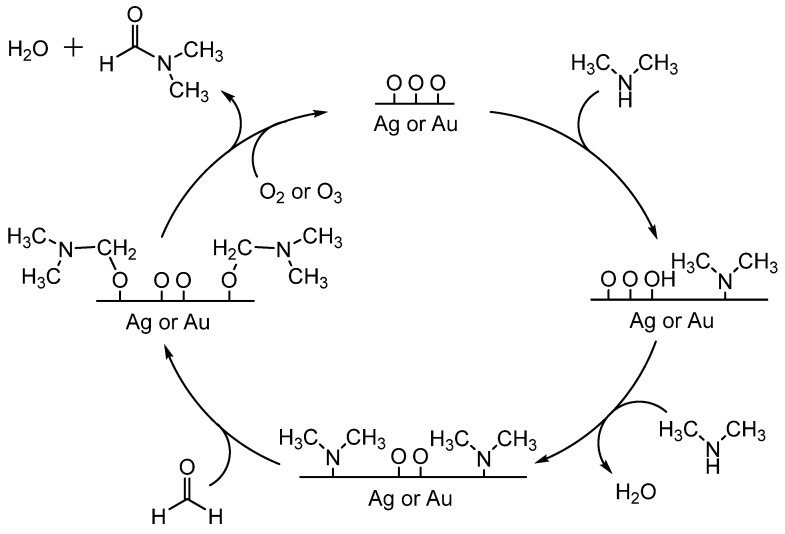
Formylation of dimethylamine with formaldehyde mediated by oxygen atoms on metallic surfaces. Scheme adapted from references [52] and [53].

Formylation of dimethylamine with formaldehyde on silver and gold surfaces was studied collaboratively by Madix and Friend [52,53]. Oxygen assisted formylation of dimethylamine on metallic silver surfaces was reported by Madix [52]. The proposed mechanistic pathway involves dissociative adsorption of O_2_ on the silver surface, along with coordination and deprotonation of the amine. When formaldehyde is introduced, it inserts into the surface-amide bond and β-hydride elimination occurs to produce the formamide (Scheme 32). A similar mechanism was proposed by Friend for gold surfaces (Scheme 32) [53]. One important distinction between these two mechanisms is that gold requires ozone (O_3_) to introduce adsorbed oxygen, while silver can employ oxygen (O_2_). These are examples in which the catalyst interacts with the amine directly during reaction with a formylating agent.

**Scheme 33:**
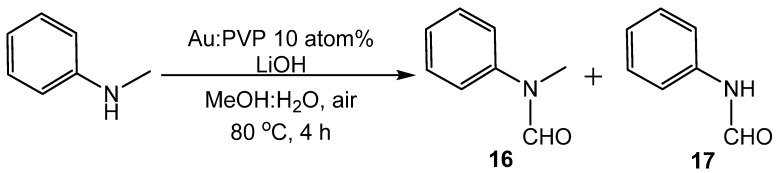
*N*-formylation of amines with methanol by nanogold particles.

Gold nanoparticles were reported by Ishida to formylate amines with methanol [54]. Formamide was produced as the primary product when Au/Al_2_O_3_ or Au/NiO was used. Gold nanoparticles were later reported by Sakurai to catalyze the formylation of amines with methanol or formaldehyde [55]. Gold nanoclusters stabilized by poly(*N*-vinyl-2-pyrrolidione) (Au:PVP) acted as the catalyst under aerobic oxidation conditions. Optimum conditions for the reaction with methanol were 10 atom% catalyst, 200 mol% LiOH as base and 1:2 methanol:water solvent at 80 °C (reflux) for 8 h (Scheme 33). When these conditions were applied to *N*-methylaniline, two products were formed: 94% yield of *N*-methylanilide **16** and 5% yield of anilide **17**. Because methanol oxidation leads to formaldehyde, formic acid, methyl formate, and carbon dioxide, control experiments with these possible formylating agents were used to ascertain which intermediate was reacting with the amine. Without methanol or a formylating agent, no reaction occurred. When formaldehyde was used in place of methanol as a 37% solution, **16** was formed in 81% yield. When either methyl formate or formic acid was used in place of methanol, no reaction occurred. Optimum conditions for this reaction when formaldehyde was used as the formyl source were 1.5 equivalents of formaldehyde, 1 atom% catalyst, 100 mol% NaOH as base and 1:2 ethanol‒water solvent at 27 °C for 9 h. Under these new conditions, the yields were best for electron-rich aromatic amines such as aniline, 4-methylaniline, *N*,4-dimethylaniline, and indoline. Sterically hindered and electron poor aromatic species produced little to no product. Primary and secondary alkyl amines also produced high yields of formamides.

**Scheme 34:**
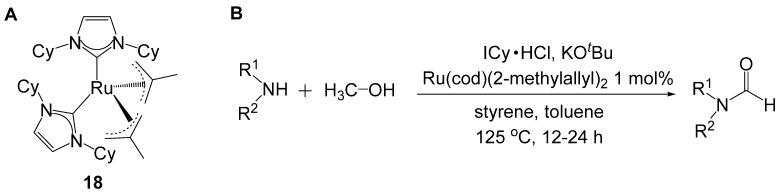
Ru-NHC catalyzed methanol activation and formylation of amines. (**A**) Catalyst **18**. (**B**) Optimum conditions.

Glorius reported *N*-formylation of amines by methanol in the presence of a ruthenium *N*-heterocyclic carbene catalyst (**18**) (Scheme 34) [56]. This complex was also reported to catalyze amide synthesis and the conditions optimized for the amide synthesis were initially applied to formylation with methanol.

When these conditions (1 mol% **18**, 1.5 equivalents of alcohol, refluxing toluene, inert atmosphere, 24 h) produced only trace amount of the formamide, the amount of methanol was increased to 3.3 equivalents. Attempted optimizations of reactant stoichiometry, concentration, solvent, and temperature did not increase the yield. When the reaction was run in a sealed container, the yield was lower and a build-up of hydrogen gas was observed. The introduction of styrene as a sacrificial hydrogen acceptor increased the conversion of starting material to formamide to 96%.

**Scheme 35:**
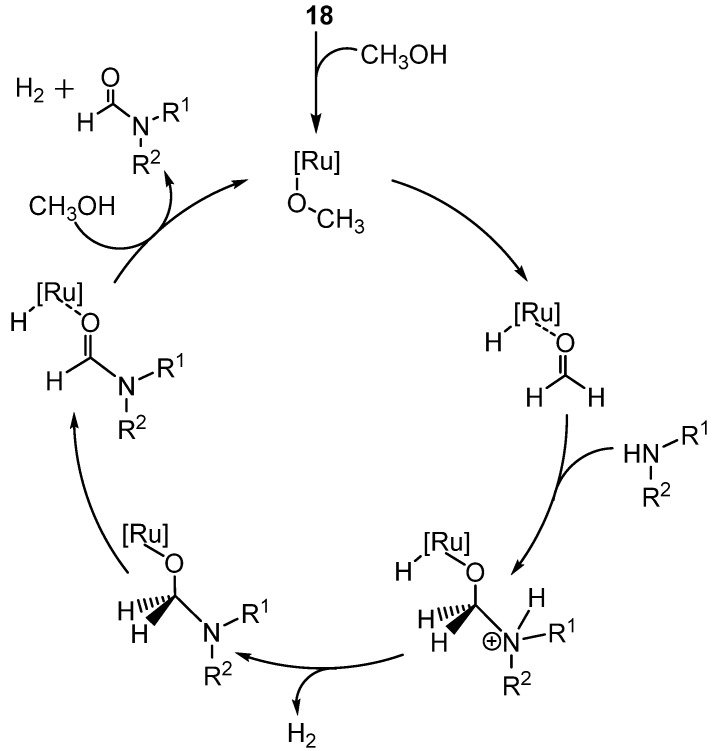
Mechanism for Ru-catalyzed formylation using methanol and **18**.

The scope was examined using primary, secondary, tertiary, and benzyl amines. Overall yields ranged from 27%–99%, with the lowest yields obtained from bulkier substrates and electron poor benzyl amines. Aromatic amines did not react. Optically pure phenylethylamine produced a 77% yield of the formamide with no loss of enantiomeric purity. During the examination of the reaction scope the catalyst **18** was formed *in situ* from the pre-catalyst Ru(cod)(2-methylallyl)_2_, the HCl salt of the NHC ligand, and base (Scheme 34). Control experiments demonstrated that the reaction was not base catalyzed. The reaction did not occur in the absence of the NHC and use of NHCs other than ICy resulted in lower conversion of starting material. Through examination of the reaction with NMR studies, a mechanism was proposed (Scheme 35). Coordination of methoxide to Ru is followed by β-hydride elimination to produce coordinated formaldehyde. The formaldehyde undergoes nucleophilic attack by the amine, then hydrogen (H_2_) is lost as the amine is deprotonated. A secondβ-hydride elimination occurs to form the formamide product, which is displaced by methanol. A second H_2_ is liberated as the original methoxide complex is formed, closing the catalytic cycle.

**Scheme 36:**
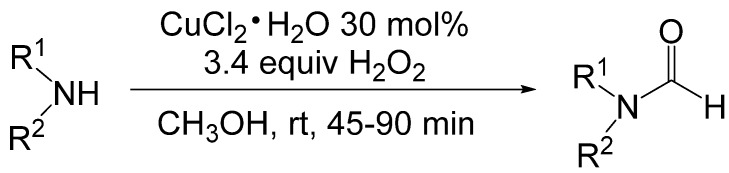
Oxidative *N*-formylation of amines with formaldehyde in the presence of copper salts.

Reddy reported formylation of primary and secondary amines by the catalytic oxidation of methanol by copper salts with hydrogen peroxide as the terminal oxidant (Scheme 36) [57]. Optimized conditions for the formylation of amines were amine, 30 mol% CuCl_2_•H_2_O and 3.4 equivalents of 6.0% w/w H_2_O_2_ in room temperature methanol for 45–90 min. Primary and secondary amines were formylated in 63%–80% yields. Slow addition of H_2_O_2_ to the reaction mixture was important for rapid formation of formylated product. When the same amount of H_2_O_2_ was added as two equal portions, decomposition of the peroxide resulted in longer reaction times and a need for additional H_2_O_2_.

## 6. Formylation by Catalytic Carbonylation

**Scheme 37:**
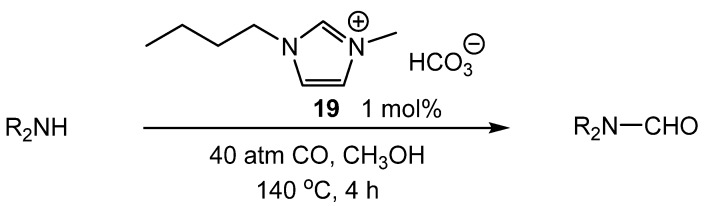
Formylation of amines using CO catalyzed by **19**.

An example of IL-catalyzed formylation using CO as the carbonyl source was reported by Lee [58]. Mixtures of the amine and IL under 40 atm CO produced formamides from primary and secondary amines in moderate to excellent yields. No urea products were observed in the reaction mixtures. Selections of IL and counterions were examined, with 1-butyl-3-methylimidazolium carbonate (**19**) exhibiting the best performance for *N*-formylation. Other optimized conditions include 40 atm CO, methanol solvent, 140 °C, and 1 mol% **19** (Scheme 37). The catalyst could be used for five trials with no loss in selectivity and only a 20% reduction in activity.

Transition metal catalysts have also been used to formylate amines using CO as the carbonyl source [59,60,61,62,63,64,65,66,67]. However, when transition metal catalysts are used, catalytic carbonylation more commonly yields ureas instead of formamides [68,69,70,71]. Formylation of amines by metal complexes and carbon monoxide was reported by Saegusa [72]. This reaction selectively formed formamides with only trace amounts of urea being observed. Various catalysts were examined, with the highest activity being obtained from CuCl. Acceleration of this reaction in water was attributed to favorable formation of the CuCl-CO complex in water. Secondary aliphatic amines proved to be better substrates for this reaction than primary aliphatic amines. Aromatic amines did not yield formamides with CuCl catalyst but they could be formylated when chloroauric acid (HAuCl_4_•H_2_O) was used as catalyst.

**Scheme 38:**
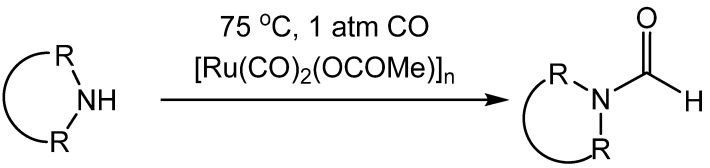
Ruthenium catalyzed carbonylation of amines.

Remple reported that the ruthenium catalyst [Ru(CO)_2_(OCOMe)]_n_. produced formamides from cyclic secondary amines using only 1 atm of carbon monoxide gas at 75 °C (Scheme 38) [61]. The mild conditions in this reaction required long reaction times (20–200 h) to ensure completion. Neither primary amines nor acyclic secondary amines could be formylated under these conditions.

Triruthenium dodecacarbonyl (Ru_3_(CO)_12_) has also been found to catalyze formylation of amines using CO as the carbonyl source [73]. Different ruthenium and rhodium catalysts were examined in this work, with the rhodium complexes producing significant amounts of urea. The best catalytic activity toward formamide production was shown by Ru_3_(CO)_12_. Optimum conditions were 0.17 mol% catalyst and 40 atm CO in benzene at 120–180 °C for 6 h. Primary aliphatic amines were successfully converted to the formamide products under these conditions. However, carbonylation of piperidine produced the formamide in only moderate yield due to competitive formation of urea.

Jenner reported that ruthenium compounds catalyzed primary and secondary amines with CO as the carbonyl source [59]. Ruthenium trichloride trihydrate (RuCl_3_•3H_2_O) showed the highest activity and selectivity towards the formation of formamides from primary amines. The cobalt catalyst Co(OAc)_2_•4H_2_O showed low conversion, and the rhodium catalyst RhCl_3_•3H_2_O showed high activity but low selectivity towards the formamide product. Using the ruthenium catalyst, primary amines were carbonylated, with the exception of the sterically crowded *tert*-butylamine. Aniline also failed to produce formamide product. Cyclic secondary amines were carbonylated to the corresponding formamides.

When acyclic secondary amines were examined, a competing pathway occurred in which transalkylation formed a tertiary amine and a primary amine from two equivalents of secondary amine. The newly formed primary amine was then carbonylated to the formamide product. Increased temperature led to lowered transalkylation but higher production of ureas. Increased pressure suppressed the reaction but did favor formamide over transalkylation products. Cobalt-ruthenium co-catalysts improved the selectivity for formylation of dialkylformamides from acyclic secondary amines over the transalkylation pathway. Using RuCl_3_•3H_2_O and Co(OAc)_2_•4H_2_O, the optimum ratio of Ru:Co for formamide production was 1.3:1.

In related work, Jenner reported the effect of solvent on carbonylation of amines with RuCl_3_•3H_2_O [60]. No correlation between the yield of formamide and dielectric constant was found. Methanol was the best solvent for ruthenium catalyzed carbonylation. Results in water were poorer, with greater yields of urea or lower conversion of amine observed. The previously examined cobalt-ruthenium catalyst, RuCl_3_•3H_2_O and Co(OAc)_2_•4H_2_O used at a ratio of 1.3:1, produced selectivity for formamide in methanol similar to that of the ruthenium catalyst. Raising the pressure to 750 atm and raising the temperature to 180 °C increased formamide selectivity with respect to urea. The best conversion and selectivity were observed when increased methanol was present. The turnover value was the highest with equal volumes of amine and methanol.

The proposed reaction pathway for this reaction was initial formation of methyl formate from methanol and CO, followed by attack from the dialkylamine forming the formamide and regenerating methanol. This reaction was successfully applied to dialkylamines and aromatic amines. However, aromatic amines still show lower conversion and selectivity than alkylamines. Sterically hindered amines such as *tert*-butylamine, which was unreactive without methanol, underwent selective formylation in the presence of ruthenium catalyst and methanol solvent.

**Scheme 39:**
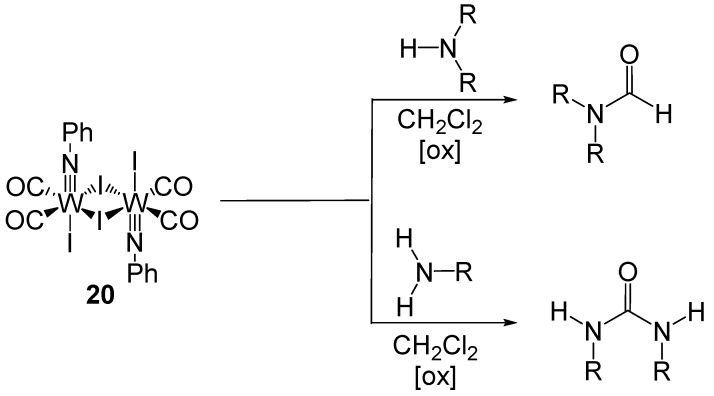
Carbonylation of amines using tungsten dimer.

McElwee-White reported tungsten complex **20** that produced formamides and ureas from secondary and primary amines respectively (Scheme 39) [63]. Using this method, secondary amines selectively form formamides in yields ranging from 8%–61% and primary amines selectively form ureas in yields ranging from 56%–105% (yields calculated per equivalent of tungsten).

**Scheme 40:**
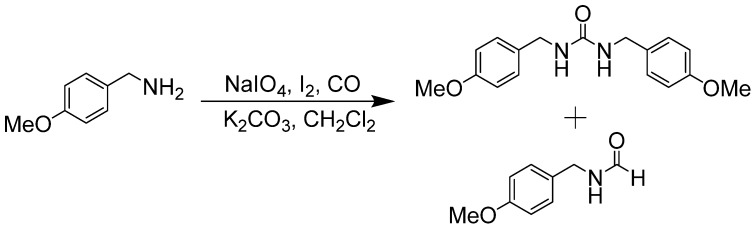
NaIO_4_-mediated carbonylation of amines to ureas and formamides.

McElwee-White later reported that W(CO)_6_ with an oxidant would carbonylate amines with CO to form ureas [74,75,76,77,78,79,80,81,82] and hydantoins [83]. During control experiments, it was discovered that a NaIO_4_/NaI oxidant/promoter system with CO in CH_2_Cl_2_ would carbonylate amines without the metal catalyst [84]. Reaction of 4-methoxybenzylamine afforded both urea and formamide products (Scheme 40).

**Scheme 41:**
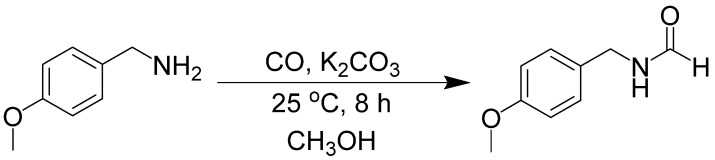
Base-mediated formylation of amines to formamides.

The NaIO_4_ mediated reaction has been optimized for formation of the formamide in methanol solvent [85]. Isotopic labeling experiments using ^13^C-labeled methanol showed that CO is the carbonyl source, not oxidized methanol. Deuterium labeling experiments revealed that the formyl hydrogen came from the acidic proton of methanol. During further investigation into this reaction, it was determined that the oxidant was unnecessary for the formylation, although it is necessary in the urea synthesis [86]. The necessary components of this reaction are amine, CO, and base (Scheme 41). The functional group tolerance of this reaction is broad. Substrates include primary, benzyl, cyclic secondary and acyclic secondary amines.

## 7. Conclusions

Methods of formylating amines include stoichiometric reagents such as chloral, formic acid, acetic formic anhydride and ammonium formate, as well as catalytic processes. Both acid catalysts and organic catalysts can be used with formic acid and formates to produce formamides. Metal catalysts can formylate amines using formic acid, paraformaldehyde, formaldehyde, and methanol as the source of the formyl moiety. Catalytic carbonylation routes produce formamides from CO in the presence of ionic liquids, transition metals, or oxidants. Base-mediated carbonylation with CO is also reported. These methods can be applied to a large scope of amines such as primary, cyclic and acyclic secondary, sterically hindered, aromatic, amino acids, and amino acid esters. Methods are available for formylation of amines with preservation of enantiomeric purity. Although there is a long history of formylation chemistry, room remains for development of new catalytic methods.
